# Supplementary material for: Financial incentives to increase stool collection rates for microbiome studies in adult bone marrow transplant patients
Source: PLoS One. 2022 May 4;17(5):e0267974. doi: 10.1371/journal.pone.0267974 (PMC9067695; doi:10.1371/journal.pone.0267974)
Supplement: Attachment — Submitted filename: PONE-D-21-40817+comments.pdf [file pone.0267974.s004.pdf]

# PLOS ONE

## Financial incentives to increase stool collection rates for microbiome studies in adult bone marrow transplant patients

--Manuscript Draft--

| | |
|---|---|
| **Manuscript Number:** | PONE-D-21-40817 |
| **Article Type:** | Research Article |
| **Full Title:** | Financial incentives to increase stool collection rates for microbiome studies in adult bone marrow transplant patients |
| **Short Title:** | Financial incentives to increase stool collection |
| **Corresponding Author:** | Anthony D Sung, MD<br>Duke University School of Medicine<br>Durham, NC UNITED STATES |
| **Keywords:** | financial incentives; stool; sample collection; hematopoietic stem cell transplantation; microbiome |
| **Abstract:** | Introduction: In order to study the role of the microbiome in hematopoietic stem cell transplantation (HCT), researchers collect stool samples from patients at various time points throughout HCT. However, stool collection requires active subject participation and may be limited by patient reluctance to handling stool.<br>Methods : We performed a prospective study on the impact of financial incentives on stool collection rates. The intervention group consisted of allogeneic HCT patients from 05/2017-05/2018 who were compensated with a $10 gas gift card for each stool sample. The intervention group was compared to a historical control group of allogeneic HCT patients from 11/2016-05/2017 who provided stool samples before the incentive was implemented. To control for possible changes in collections over time, we also compared a contemporaneous control group of autologous HCT patients from 05/2017-05/2018 with a historical control group of autologous HCT patients from 11/2016-05/2017; neither autologous HCT group was compensated. The collection rate was defined as the number of samples provided divided by the number of time points we attempted to obtain stool.<br>Results: There were 35 allogeneic HCT patients in the intervention group, 19 allogeneic HCT patients in the historical control group, 142 autologous HCT patients in the contemporaneous control group (that did not receive a financial incentive), and 75 autologous HCT patients in the historical control group. Allogeneic HCT patients in the intervention group had significantly higher average overall collection rates when compared to the historical control group allogeneic HCT patients (80% vs 37%, p<0.0001). There were no significant differences in overall average collection rates between the autologous HCT patients in the contemporaneous control and historical control groups (36% vs 32%, p=0.2760).<br>Conclusion : Our results demonstrate that a modest incentive can significantly increase collection rates. These results may help to inform the design of future studies involving stool collection. |
| **Order of Authors:** | Jillian C. Thompson |
| | Yi Ren |
| | Kristi Romero |
| | Meagan Lew |
| | Amy Bush |
| | Julia A. Messina |
| | Sin-Ho Jung |
| | Sharareh Siamakpour-Reihani |
| | Julie Miller |
| | Robert R. Jenq |
| | |

Powered by Editorial Manager® and ProduXion Manager® from Aries Systems Corporation

| | |
|---|---|
| | Jonathan U. Peled |
| | Marcel R.M. van den Brink |
| | Nelson J. Chao |
| | Mark G. Shrime |
| | Anthony D. Sung |
| **Additional Information:** | |
| **Question** | **Response** |
| **Financial Disclosure**<br><br>Enter a financial disclosure statement that describes the sources of funding for the work included in this submission. Review the submission guidelines for detailed requirements. View published research articles from *PLOS ONE* for specific examples.<br><br>This statement is required for submission and **will appear in the published article** if the submission is accepted. Please make sure it is accurate.<br><br>**Unfunded studies**<br>Enter: *The author(s) received no specific funding for this work.*<br><br>**Funded studies**<br>Enter a statement with the following details:<br>• Initials of the authors who received each award<br>• Grant numbers awarded to each author<br>• The full name of each funder<br>• URL of each funder website<br>• Did the sponsors or funders play any role in the study design, data collection and analysis, decision to publish, or preparation of the manuscript?<br>• **NO** - Include this sentence at the end of your statement: *The funders had no role in study design, data collection and analysis, decision to publish, or preparation of the manuscript.*<br>• **YES** - Specify the role(s) played.<br><br>* typeset | This study was supported by P30-AG028716-13 Mini#6 (to ADS), R01 HL124112 (to RRJ, ADS), R01-CA203950 (to ADS, NJC), Seres (to ADS), and the ASH Scholar Award (to ADS). The funders had no role in study design, data collection and analysis, decision to publish, or preparation of the manuscript. |
| **Competing Interests**<br><br>Use the instructions below to enter a competing interest statement for this submission. On behalf of all authors, | I have read the journal's policy and the authors of this manuscript have the following competing interests:<br><br>MRM receives funding from Seres, Evelo, Jazz Pharmaceuticals, Therakos, Amgen, Merck & Co, Inc, Magenta Therapeutics, Smart Immune, Juno, and serves on the |

| | |
|---|---|
| disclose any competing interests that could be perceived to bias this work—acknowledging all financial support and any other relevant financial or non-financial competing interests.<br><br>This statement is required for submission and **will appear in the published article** if the submission is accepted. Please make sure it is accurate and that any funding sources listed in your Funding Information later in the submission form are also declared in your Financial Disclosure statement.<br><br>View published research articles from *PLOS ONE* for specific examples.<br><br>**NO authors have competing interests**<br><br>Enter: *The authors have declared that no competing interests exist.*<br><br>**Authors with competing interests**<br><br>Enter competing interest details beginning with this statement:<br><br>*I have read the journal's policy and the authors of this manuscript have the following competing interests: [insert competing interests here]*<br><br>* typeset | DKMS Advisory Board. ADS receives funding from Novartis, Merck, Seres, and serves as a consultant to AVROBIO. There is no overlap between ADS' work with Novartis, Merck, AVROBIO, and this project. While Seres did fund the collection of stool samples, which led to some of the data presented, they had no role in data analysis and interpretation. Other than the competing interests statement, none of these companies will be mentioned by name. |
| **Ethics Statement**<br><br>Enter an ethics statement for this submission. This statement is required if the study involved:<br><br>• Human participants<br>• Human specimens or tissue<br>• Vertebrate animals or cephalopods<br>• Vertebrate embryos or tissues<br>• Field research<br><br>Write "N/A" if the submission does not require an ethics statement. | This study was approved by the Duke Institutional Review Board, and written informed consent was obtained from all study participants. |

Powered by Editorial Manager® and ProduXion Manager® from Aries Systems Corporation

| | |
|---|---|
| General guidance is provided below. Consult the submission guidelines for detailed instructions. **Make sure that all information entered here is included in the Methods section of the manuscript.**<br><br>**Format for specific study types**<br><br>**Human Subject Research (involving human participants and/or tissue)**<br>• Give the name of the institutional review board or ethics committee that approved the study<br>• Include the approval number and/or a statement indicating approval of this research<br>• Indicate the form of consent obtained (written/oral) or the reason that consent was not obtained (e.g. the data were analyzed anonymously)<br><br>**Animal Research (involving vertebrate animals, embryos or tissues)**<br>• Provide the name of the Institutional Animal Care and Use Committee (IACUC) or other relevant ethics board that reviewed the study protocol, and indicate whether they approved this research or granted a formal waiver of ethical approval<br>• Include an approval number if one was obtained<br>• If the study involved *non-human primates*, add *additional details* about animal welfare and steps taken to ameliorate suffering<br>• If anesthesia, euthanasia, or any kind of animal sacrifice is part of the study, include briefly which substances and/or methods were applied<br><br>**Field Research**<br><br>Include the following details if this study involves the collection of plant, animal, or other materials from a natural setting:<br>• Field permit number<br>• Name of the institution or relevant body that granted permission | |
| **Data Availability**<br><br>Authors are required to make all data | Yes - all data are fully available without restriction |

| | |
|---|---|
| underlying the findings described fully available, without restriction, and from the time of publication. PLOS allows rare exceptions to address legal and ethical concerns. See the PLOS Data Policy and FAQ for detailed information.<br><br>A Data Availability Statement describing where the data can be found is required at submission. Your answers to this question constitute the Data Availability Statement and **will be published in the article**, if accepted.<br><br>**Important:** Stating 'data available on request from the author' is not sufficient. If your data are only available upon request, select 'No' for the first question and explain your exceptional situation in the text box.<br><br>Do the authors confirm that all data underlying the findings described in their manuscript are fully available without restriction? | |
| **Describe where the data may be found in full sentences. If you are copying our sample text, replace any instances of XXX with the appropriate details.**<br><br>• If the data are **held or will be held in a public repository**, include URLs, accession numbers or DOIs. If this information will only be available after acceptance, indicate this by ticking the box below. For example: *All XXX files are available from the XXX database (accession number(s) XXX, XXX.*).<br>• If the data are all contained **within the manuscript and/or Supporting Information files**, enter the following: *All relevant data are within the manuscript and its Supporting Information files.*<br>• If neither of these applies but you are able to provide **details of access elsewhere**, with or without limitations, please do so. For example:<br><br>*Data cannot be shared publicly because of [XXX]. Data are available from the* | All relevant data are within the manuscript and its Supporting Information files. |

| | |
|---|---|
| *XXX Institutional Data Access / Ethics Committee (contact via XXX) for researchers who meet the criteria for access to confidential data.*<br><br>*The data underlying the results presented in the study are available from (include the name of the third party and contact information or URL).*<br>• This text is appropriate if the data are owned by a third party and authors do not have permission to share the data.<br><br>* typeset | |
| Additional data availability information: | |

Powered by Editorial Manager® and ProduXion Manager® from Aries Systems Corporation

Manuscript

Click here to access/download;Manuscript;Financial Incentives in Stool Collection PLOS ONE.docx

**Title:** Financial incentives to increase stool collection rates for microbiome studies in adult bone marrow transplant patients

**Short Title:** Financial incentives to increase stool collection

**Authors and Affiliations:**

Jillian C. Thompson[1], Yi Ren[2], Kristi Romero[1], Meagan Lew[1], Amy Bush[1], Julia A. Messina[3], Sin-Ho Jung[2], Sharareh Siamakpour-Reihani[1], Julie Miller[4], Robert R. Jenq[5], Jonathan U. Peled[6], Marcel R. M. van den Brink[6], Nelson J. Chao[1], Mark G. Shrime[7¶], Anthony D. Sung[1*¶]

[1]Division of Hematologic Malignancies and Cellular Therapy, Duke University School of Medicine, Durham, North Carolina, United States of America

[2]Department of Biostatistics and Bioinformatics, Duke University School of Medicine, Durham, North Carolina, United States of America

[3]Division of Infectious Diseases, Duke University School of Medicine, Durham, North Carolina, United States of America

[4]Center for Advanced Hindsight, Duke University School of Medicine, Durham, North Carolina, United States of America

[5]Departments of Genomic Medicine and Stem Cell Transplantation Cellular Therapy, MD Anderson Cancer Center, University of Texas, Houston, Texas, United States of America

[6]Department of Medicine, Adult Bone Marrow Transplant Service, Memorial Sloan Kettering Cancer Center, New York, New York, United States of America

[7]Department of Otolaryngology Head and Neck Surgery, Harvard Medical School, Boston, Massachusetts, United States of America

*Corresponding Author

E-mail: anthony.sung@duke.edu (AS)

¶These authors contributed equally to the work.

# Abstract

**Introduction:** In order to study the role of the microbiome in hematopoietic stem cell transplantation (HCT), researchers collect stool samples from patients at various time points throughout HCT. However, stool collection requires active subject participation and may be limited by patient reluctance to handling stool.

**Methods**: We performed a prospective study on the impact of financial incentives on stool collection rates. The intervention group consisted of allogeneic HCT patients from 05/2017-05/2018 who were compensated with a $10 gas gift card for each stool sample. The intervention group was compared to a historical control group of allogeneic HCT patients from 11/2016-05/2017 who provided stool samples before the incentive was implemented. To control for possible changes in collections over time, we also compared a contemporaneous control group of autologous HCT patients from 05/2017-05/2018 with a historical control group of autologous HCT patients from 11/2016-05/2017; neither autologous HCT group was compensated. The collection rate was defined as the number of samples provided divided by the number of time points we attempted to obtain stool.

**Results:** There were 35 allogeneic HCT patients in the intervention group, 19 allogeneic HCT patients in the historical control group, 142 autologous HCT patients in the contemporaneous control group (that did not receive a financial incentive), and 75 autologous HCT patients in the historical control group. Allogeneic HCT patients in the intervention group had significantly higher average overall collection rates when compared to the historical control group allogeneic HCT patients (80% vs 37%, $p<0.0001$). There were no significant differences in overall average

collection rates between the autologous HCT patients in the contemporaneous control and historical control groups (36% vs 32%, p=0.2760).

**Conclusion**: Our results demonstrate that a modest incentive can significantly increase collection rates. These results may help to inform the design of future studies involving stool collection.

# Introduction

The human gut microbiome is the myriad of bacteria, archaea, viruses, and fungi that reside in the human gastrointestinal tract.[1-3] In hematopoietic stem cell transplantation (HCT), disruption of the gut microbiome, concomitant of the transplant conditioning regimen, is associated with post-transplant complications such as the development of graft-versus-host disease and infections.[4, 5] Although many strides have been made in investigating the complex relationship between the gut microbiome and its host, further elucidation of the role of the microbiome in patients undergoing HCT is essential in order to improve patient outcomes.[1] The gut microbiome can be studied with next-generation sequencing of microbial nucleic acids that are extracted from human stool samples.[2, 6]

Despite knowing how to utilize human stool samples to investigate the microbiome, we have found that the challenge lies in collecting enough stool samples from study participants at various time points throughout the transplant process. Paramsothy et al. found that this challenge exists even when requesting stool samples from healthy donors, demonstrating that approximately 40% of potential donors declined to participate in their study due to the burden of providing stool samples over a six-week period.[7] A different study focused on at-home stool

collection, specifically in cancer patients, by Hogue et al. revealed that only 58% of consented patients provided baseline stool samples and only 25% of consented patients provided follow-up stool samples.[8] Unlike other human tissue sampling methods such as drawing blood or swabbing the skin, collecting stool involves more effort on behalf of the patients, especially in the outpatient setting where patients must handle the stool themselves before subsequently placing in a specimen cup.[9] Thus, stool collection compliance in research studies may be hindered as a result of patient apprehension to handling stool due to factors such as embarrassment, disgust, and privacy concerns. [10-12] Furthermore, cancer patients may experience weakness or constipation due to treatment, which can result in noncompliance with stool collection protocols.[8]

However, financial incentives may motivate patients to be more willing to provide stool samples, thus leading to increased adherence to study protocols. For example, Green et al. found that both a modest incentive of $10 and a probabilistic incentive of a 10% chance of winning $50 significantly increased rates of another stool-related research activity, fecal immunochemical testing ($10 incentive 73.3% vs 66.2%, p=0.04; chance of winning 71.8% vs 66.2%, p=0.04), despite not increasing colorectal cancer screening via colonoscopy. [9, 13] Incorporating a strategy that includes financial incentives into a research study design can significantly increase the desired outcome. [14-16] Therefore, we believed that we could significantly improve study participant compliance to providing stool samples throughout the HCT process by giving them compensation for their stool samples.

# Materials and methods

This study was approved by the Duke Institutional Review Board, and written informed consent was obtained from all study participants.

## Defining groups and sample collection

Patients in the intervention group were compensated financially for their stool samples. The intervention group was composed of patients undergoing allogeneic HCT with treatment start dates between 05/11/2017, the date when the financial incentive was implemented, and 05/11/2018. Collection rates, in addition to baseline characteristics, of the allogeneic HCT patients in the intervention group were compared to those of allogeneic HCT patients from a historical control group. The historical control allogeneic HCT patients had treatment start dates between 11/10/2016, the date a study team member started actively managing stool collection in patients through distribution of collection coolers and consistent follow-up, and 05/10/2017. The allogeneic HCT patients in the historical control group were not compensated.

In order to control for potential differences in stool collection over two different time periods, a contemporaneous control group was also included in the study design. The contemporaneous control group consisted of patients undergoing autologous HCT with treatment start dates between 05/11/2017 and 05/11/2018 who were not compensated in any way for their stool samples. Collection rates and baseline characteristics of the autologous HCT patients in the contemporaneous control group were compared to those of autologous HCT patients from a historical control group. The historical control autologous HCT patients had treatment start dates between 11/10/2016 and 05/10/2017, and these patients were not compensated in any way for

their stool samples. Of note, no autologous HCT patients, regardless of control group, were compensated in this study; only the allogeneic HCT patients from the intervention group were compensated. Regardless of group, if a patient's HCT treatment start date fell outside of the specified date ranges for a group, this patient was not included in the final analysis in order to prevent overlap between groups.

In this prospective cohort study, allogeneic HCT patients in both the intervention group and the historical control group were required to provide stool samples at the following time points throughout the HCT process: pre-HCT, day 0 (the day of HCT), and days 7, 14, 21, 30, 60, and 90 post-HCT. Since autologous HCT patients do not come to the Adult Blood and Marrow Transplant Clinic as frequently as allogeneic HCT patients, autologous HCT patients in both the contemporaneous control group and the historical control group were only required to provide stool samples at the following time points throughout the HCT process: pre-HCT and days 7, 14, and 90 post-HCT. Figure 1 provides an overview of the study, depicting group comparisons and when samples were collected from each group.

**Fig 1. Sample Collection Timeline for All Groups.** Allo Collection Schedule: Pre-HCT → Day 0 → Day 7 → Day 14 → Day 21 → Day 30 → Day 60 →Day 90
Auto Collection Schedule: Pre-HCT → Day 7 → Day 14 → Day 90

Stool samples were categorized as "inpatient" if scheduled to be provided by the patient while admitted to the hospital at the time of sample collection or "outpatient" if not admitted at the time of sample collection. When samples were collected in the outpatient setting, the patient was provided with a stool collection kit comprised of a stool collection hat, a specimen cup, a tongue depressor, and a pair of gloves, along with a cooler and ice pack to store the sample after collecting it themselves. In the inpatient setting, nurses provided patients with a stool collection

hat, but the nurses were the ones that performed all the steps of collection and storage after defecation. Patients in the intervention group were allocated a $10 gas gift card for each stool sample provided regardless of whether stool was collected in the inpatient or outpatient setting.

# Data collection

Stool collection was tracked by assessing the number of samples given at their required time points. A collection probability was delineated as the number of samples actually provided by the participant divided by the number of time points for which we required samples be provided. The Duke Adult Blood and Marrow Transplant database was used to query the exact dates that stool samples were collected from each patient in order to verify that samples were provided at the required time points. Only stool samples given between 05/11/2017 and 05/11/2018 were accounted for when determining collection rates for both the intervention and contemporaneous control groups whereas only stool samples given between 11/10/2016 and 05/10/2017 were accounted for when determining collection rates for the historical control group. If a sample was given outside of these time frames, the sample was not included when determining the collection rate. Thus, if a time point typically requiring a sample be given fell outside of these time frames, that time point was not included when assessing compliance, neither hurting the participant's collection rate if no sample was given, nor helping the participant if a sample was given. Furthermore, if a participant withdrew from the study or died, then the subsequent time points after date of death or withdrawal were not included in the analysis. Each sample was tracked for whether it was provided in the inpatient or outpatient setting in order to assess inpatient and outpatient collection rates. Demographic data such as age, gender, race, ethnicity, disease, and conditioning type were abstracted from the Duke Adult Blood and Marrow Transplant database and from electronic medical records.

## Statistical analysis

Baseline demographics were summarized with N (%) for categorical variables and median (interquartile range) with mean (standard deviation) for continuous variables for all patients. Chi-square tests or Fisher's exact tests were utilized to compare categorical variables, as appropriate, and Wilcoxon Rank Sum tests or t-tests were utilized to compare continuous variables, as appropriate. For allogeneic patients, negative binomial regression with generalized estimating equation (GEE) was performed to model the inpatient and outpatient collection rates of each patient, if applicable, and GEE with compound symmetry correlation structure was used to account for the correlation of the two rates for each patient. Other covariates such as age, gender, race, disease, and conditioning type were adjusted for in order to avoid confounding. All analyses were conducted using SAS version 9.4 (SAS Institute, Cary, NC) and R version 3.5.0.

# Results

Fifty-four patients undergoing allogeneic HCT and 217 patients undergoing autologous HCT were included in the study cohort. Of the 54 allogeneic HCT patients, there were 35 (64.8%) allogeneic HCT patients in the intervention group that were compared to 19 (35.2%) allogeneic HCT patients in the historical control group. Although not significantly different, the intervention group tended to be slightly older at transplant (61 vs 51 median age, p=0.0853) and included a smaller proportion of female patients (28.6% vs 52.6%, p=0.0804). There were also no significant differences between the two groups of allogeneic HCT patients with regard to other baseline demographics such as race, ethnicity, disease, and conditioning (Table 1.)

**Table 1. Baseline Allogeneic HCT Patient Characteristics**

| | Intervention Group | Historical Control Group | All Patients | |
|---|---|---|---|---|
| | N=35 (64.8%) | N=19 (35.2%) | N=54 (100%) | P-Value |
| Age at Transplant, median (IQR)* | 61 (50 - 64) | 51 (35 - 59) | 56 (46 - 63) | 0.0853 |
| Gender, female, no. (%)** | 10 (28.6%) | 10 (52.6%) | 20 (37%) | 0.0804 |
| Race, no. (%) | | | | |
| Black/African American | 2 (5.7%) | 4 (21.1%) | 6 (11.1%) | 0.2693 |
| Other/Unknown | 2 (5.7%) | 0 (0%) | 2 (3.7%) | |
| White | 31 (88.6%) | 15 (78.9%) | 46 (85.2%) | |
| Ethnicity, no. (%) | | | | |
| Hispanic or Latino | 1 (2.9%) | 1 (5.3%) | 2 (3.7%) | 1.0000 |
| Not Hispanic or Latino | 33 (94.3%) | 18 (94.7%) | 51 (94.4%) | |
| Unknown | 1 (2.9%) | 0 (0%) | 1 (1.9%) | |
| Disease, no. (%) | | | | |
| Acute Leukemia | 13 (37.1%) | 8 (42.1%) | 21 (38.9%) | 0.4396 |
| Lymphoma | 4 (11.4%) | 4 (21.1%) | 8 (14.8%) | |
| MDS/MPN | 14 (40%) | 4 (21.1%) | 18 (33.3%) | |
| Multiple Myeloma | 1 (2.9%) | 2 (10.5%) | 3 (5.6%) | |
| Other | 3 (8.6%) | 1 (5.3%) | 4 (7.4%) | |
| Myeloablative Conditioning, no. (%) | 23 (65.7%) | 13 (68.4%) | 36 (66.7%) | 0.8403 |

*t-test was used to test age difference and Wilcoxon Rank Sum tests were used for other continuous variables.

**Chi-squared test was used to test gender difference and Fisher's exact tests were used for other categorical variables.

Of the 217 autologous HCT patients, there were 142 (65.3%) autologous HCT in the contemporaneous control group that were compared to 75 (34.7%) autologous HCT patients in the historical control group. The majority of patients in both groups received autologous HCT to treat multiple myeloma. There were no significant differences between the two groups of autologous HCT patients with regard to baseline demographics such as age at transplant, gender, race, ethnicity, and disease (Table 2).

**Table 2. Baseline Autologous HCT Patient Characteristics**

| | **Contemporaneous Control Group** | **Historical Control Group** | **All Patients** | |
|---|---|---|---|---|
| | **N=142 (65.3%)** | **N=75 (34.7%)** | **217 (100%)** | **P-Value** |
| Age at Transplant, median (IQR)* | 60 (53 - 67) | 62 (53 - 67) | 61 (53 - 67) | 0.6255 |
| Gender, female, no. (%)** | 55 (38.7%) | 35 (46.7%) | 90 (41.5%) | 0.2592 |
| Race, no. (%) | | | | |
| Black/African American | 31 (21.8%) | 19 (25.3%) | 50 (23%) | 0.6697 |
| Other/Unknown | 7 (4.9%) | 5 (6.7%) | 12 (5.5%) | |
| White | 104 (73.2%) | 51 (68%) | 155 (71.4%) | |
| Ethnicity, no. (%) | | | | |
| Hispanic or Latino | 3 (2.1%) | 2 (2.7%) | 5 (2.3%) | 0.2176 |
| Not Hispanic or Latino | 138 (97.2%) | 70 (93.3%) | 208 (95.9%) | |
| Unknown | 1 (0.7%) | 3 (4%) | 4 (1.8%) | |
| Disease, no. (%) | | | | |
| Acute Leukemia | 1 (0.7%) | 0 (0%) | 1 (0.5%) | 0.9319 |
| Lymphoma | 39 (27.5%) | 18 (24%) | 57 (26.3%) | |
| Multiple Myeloma | 96 (67.6%) | 54 (72%) | 150 (69.1%) | |
| Other | 6 (4.2%) | 3 (4%) | 9 (4.1%) | |

*t-test was used to test age difference and Wilcoxon Rank Sum tests were used for other continuous variables.

**Chi-squared test was used to test gender difference and Fisher's exact tests were used for other categorical variables.

The allogeneic HCT patients in the intervention group displayed better compliance to stool collection protocols than the allogeneic HCT patients in the historical control group (Table 3). For instance, the mean overall collection rate in the intervention group of allogeneic HCT patients was much higher than the mean overall collection rate of the allogeneic HCT patients in the historical control group (80% vs 37%, p<0.0001). In addition to an increased mean overall collection rate, the allogeneic HCT patients in the intervention group also demonstrated significantly increased mean outpatient collection rates (84% vs 23%, p<0.0001) and significantly increased mean inpatient collection rates (71% vs 46%, p=0.0409).

**Table 3. Allogeneic HCT Patient Stool Collection Rates**

| | **Intervention Group** | **Historical Control Group** | **All Patients** | |
|---|---|---|---|---|
| | **N=35 (64.8%)** | **N=19 (35.2%)** | **N=54 (100%)** | **P-Value** |
| Overall Collection Rate | | | | |
| Median (IQR) | 0.875 (0.75 - 1) | 0.375 (0 - 0.67) | 0.75 (0.375 - 0.875) | <.0001 |
| Mean (SD) | 0.80 (0.24) | 0.37 (0.36) | 0.65 (0.35) | |
| Outpatient Collection Rate | | | | |
| Median (IQR) | 1 (0.8 - 1) | 0 (0 - 0.5) | 0.82 (0.25 - 1) | <.0001 |
| Mean (SD) | 0.84 (0.27) | 0.23 (0.33) | 0.64 (0.41) | |
| Inpatient Collection Rate | | | | |
| Median (IQR) | 0.8 (0.5 - 1) | 0.5 (0 - 0.75) | 0.75 (0.4 - 1) | 0.0409 |

| | Intervention Group | Historical Control Group | All Patients | |
|---|---|---|---|---|
| | **N=35 (64.8%)** | **N=19 (35.2%)** | **N=54 (100%)** | **P-Value** |
| Mean (SD) | 0.71 (0.36) | 0.46 (0.41) | 0.62 (0.40) | |

*Wilcoxon Rank Sum tests were used to test the rate differences.

On the other hand, there were no differences in compliance to stool collection protocols between the autologous patients in the contemporaneous control and historical control groups (Table 4.) Mean overall collection rates were similar in both groups of autologous patients (36% vs 32%, p=0.2760). Furthermore, mean outpatient collection rates (30% vs 28%, p=0.5360) and mean inpatient collection rates (46% vs 59%, p=0.2509) were comparable as well. Figure 2a demonstrates the proportion of stool samples collected at each time point in the outpatient setting, whereas Figure 2b demonstrates the proportion of stool samples collected in the inpatient setting, amongst the allogeneic and autologous transplant patients in the intervention and control groups.

**Table 4. Autologous HCT Patient Stool Collection Rates**

| | Contemporaneous Control Group | Historical Control Group | All Patients | |
|---|---|---|---|---|
| | **N=142 (65.3%)** | **N=75 (34.7%)** | **217 (100%)** | **P-Value** |
| Overall Collection Rate | | | | |
| Median (IQR) | 0.25 (0 - 0.75) | 0.25 (0 - 0.5) | 0.25 (0 - 0.67) | 0.2760 |
| Mean(SD) | 0.36 (0.35) | 0.32 (0.37) | 0.35 (0.36) | |
| Outpatient Collection Rate | | | | |
| Median (IQR) | 0 (0 - 0.67) | 0 (0 - 0.5) | 0 (0 - 0.67) | 0.5360 |
| Mean(SD) | 0.30 (0.36) | 0.28 (0.38) | 0.29 (0.37) | |
| Inpatient Collection Rate | | | | |
| Median (IQR) | 0.5 (0 - 1) | 1 (0 - 1) | 0.5 (0 - 1) | 0.2509 |

| | Contemporaneous Control Group | Historical Control Group | All Patients | |
|---|---|---|---|---|
| | N=142 (65.3%) | N=75 (34.7%) | 217 (100%) | P-Value |
| Mean(SD) | 0.46 (0.47) | 0.59 (0.50) | 0.49 (0.47) | |

**Fig 2a. Outpatient Collections across Time Points for All Groups.** Each collection time point is indicated at the top of the figure: Pre, T+0 (Day 0), T+ 1wk (Day 7), T+ 2wk (Day 14), T+ 3wk (Day 21), T+ 30d (Day 30), T+ 60d (Day 60), T+ 90d (Day 90). At each time point, the proportion of samples collected/not collected are shown for each group. If denoted as 'collected' (represented in black), this proportion of samples was successfully provided. If denoted as 'not collected' (represented in dark gray), this proportion of samples was not provided. If denoted as 'NA' (represented in light gray), this time point was not a required collection time point for that particular group.

**Fig 2b. Inpatient Collections across Time Points for All Groups.** Each collection time point is indicated at the top of the figure: Pre, T+0 (Day 0), T+ 1wk (Day 7), T+ 2wk (Day 14), T+ 3wk (Day 21), T+ 30d (Day 30), T+ 60d (Day 60), T+ 90d (Day 90). At each time point, the proportion of samples collected/not collected are shown for each group. If denoted as 'collected' (represented in black), this proportion of samples was successfully provided. If denoted as

‘not collected’ (represented in dark gray), this proportion of samples was not provided. If denoted as ‘NA’ (represented in light gray), this time point was not a required collection time point for that particular group or, in the case of the day 90 time point, none of the samples were provided in the inpatient setting at this time point.

Table 5 displays a multivariate analysis modeling sample collection rates amongst the allogeneic transplant patients. The stool sample collection rate was 3.853 times higher in the intervention group than the stool sample collection rate in the historical control group (95% CI: 1.938, 7.657). There were no overall significant differences in sample collection rates after adjusting for covariates such as age, gender, conditioning, race, and disease. However, allogeneic transplant patients with lymphoma, MDS/MPN, or multiple myeloma had significantly higher incidence rate ratios for sample collection rates when compared to allogeneic transplant patients with acute leukemia. Furthermore, African American allogeneic transplant patients had 2.658 times higher stool sample collection rates when compared to white allogeneic transplant patients (95% CI: 1.36, 5.194).

**Table 5. Negative Binomial Regression with GEE on Stool Sample Collection Rate of Allogeneic Transplant Patients**

| | **Incident Rate Ratio (95% CI)** | **P-Value** | **Overall P-Value** |
|---|---|---|---|
| Group | | | |
| Historical Control Group | -REF- | | 0.001 |
| Intervention Group | 3.853 (1.938 - 7.657) | <0.001 | |
| Age | | | |
| Continuous | 0.987 (0.971 - 1.003) | 0.112 | |
| Gender | | | |
| Male | -REF- | | 0.365 |
| Female | 0.756 (0.431 - 1.328) | 0.331 | |
| Conditioning | | | |

| | | | |
|---|---|---|---|
| Myeloablative | -REF- | | 0.140 |
| NMA/RIC | 0.603 (0.321 - 1.132) | 0.115 | |
| Race | | | |
| White | -REF- | | 0.098 |
| Black/African American | 2.658 (1.36 - 5.194) | 0.004 | |
| Other/Unknown | 0.967 (0.398 - 2.35) | 0.942 | |
| Disease | | | |
| Acute Leukemia | -REF- | | 0.068 |
| Lymphoma | 2.345 (0.938 - 5.863) | 0.068 | |
| MDS/MPN | 1.84 (1.039 - 3.258) | 0.036 | |
| Multiple Myeloma | 5.146 (1.993 - 13.287) | <0.001 | |
| Other | 0.625 (0.183 - 2.134) | 0.454 | |

# Discussion

With a significant increase in overall, outpatient, and inpatient collection rates in the intervention group, our results indicate that even moderate incentivization in the form of a $10 gas gift card can be efficacious in improving stool collection compliance in research. While this stands in contrast to other studies of $5-$20 incentives that showed no increase in collection rates of at-home fecal immunochemical testing or fecal occult blood testing, we believe our study demonstrates that a modest financial incentive of $10 for each stool sample is effective in procuring higher rates of stool samples for a couple of possible reasons.[17-19] For instance, the serial collection design of the study, requiring stool samples at multiple time points, provides a study participant in the intervention group multiple opportunities to earn a $10 gas gift card for each stool sample, thus the potential to actually earn more than $10 in gas gift cards during the entirety of the study. Another possible contributing factor for the effectiveness of financial incentives in our study was that study participants had the opportunity to return their required

stool samples in-person at their clinic appointments, avoiding having to mail the sample which may be perceived by some as an additional inconvenience. Furthermore, employing a contemporaneous control group that did not receive the financial incentive into the study design addresses the possible confounders associated with potential discrepancies in stool collection rates over time, strengthening our finding that the increase in collection rates can be attributed to the financial incentive.

Despite the effectiveness of the financial incentive, our study is not without limitations. For instance, although accounted for in the statistical analyses, there are considerable differences in sample size between not only the comparison groups within each transplant type, but also between the total number of allogeneic and autologous transplant patients included in the study. The difference in the number of allogeneic and autologous transplant study participants is reflective of our patient population: about twice as many adult autologous stem cell transplants are performed each year than adult allogeneic transplants at Duke. Another limitation of the study is that the financial incentive was only made available to allogeneic transplant patients due to funding restraints; this was accounted for by only performing comparisons within the same transplant type. The non-randomization of the study is also a limiting factor because it does not take into account confounders such as social determinants of health that may make someone more or less inclined to participate in a research study involving financial incentives. Furthermore, although it was found that African American allogeneic transplant patients had higher stool sample collection rates when compared to white allogeneic transplant patients, there is a lack of racial and ethnic diversity in this study with the majority of study participants being non-Hispanic whites.

Effort on behalf of the patient is required most when providing a stool sample in the

outpatient setting since patients must do the collection process themselves, as opposed to the inpatient setting where nursing staff aid with stool collection for admitted patients. Thus, the formidable boost in collection rates in the outpatient setting in the intervention group underscore the role of the financial incentive in this study. While the increase in inpatient collection rates in the intervention group is still significant, the average inpatient collection rate associated with the intervention group is mediated in the part by the role of nurses who work with patients to collect samples in that setting. Also, inpatient collection time points may have been missed when patients were only admitted for 24-48 hours for indications such as febrile neutropenia before being discharged to continue antibiotics in the outpatient setting, thus leaving a very narrow window for inpatient collection.

While this study was performed in a specialized HCT patient population, this study design utilizing financial incentives to increase stool collection rates may be able to be executed in a myriad of patient populations. If these results are generalizable, other researchers attempting to procure stool samples for microbiome studies may be able to increase their patient compliance and improve their stool collection rates. Future directions for this study will be to observe the use of financial incentives for stool collection in the HCT population longitudinally in order to evaluate whether the effectiveness of the financial incentive would wear off over time. Furthermore, with more funding, autologous HCT patients can be included in the study. Another next step is to investigate how social determinants of health affect stool collection rates in the HCT population, identifying how factors such as socioeconomic status influence compliance and willingness to participate in a study utilizing financial incentives.

# References


1. Andermann TM, Peled JU, Ho C, Reddy P, Riches M, Storb R, et al. The Microbiome and Hematopoietic Cell Transplantation: Past, Present, and Future. Biology of Blood and Marrow Transplantation. 2018;24(7):1322-40.

2. Zama D, Biagi E, Masetti R, Gasperini P, Prete A, Candela M, et al. Gut microbiota and hematopoietic stem cell transplantation: where do we stand? Bone Marrow Transplantation. 2016;52:7.

3. Abrahamson M, Hooker E, Ajami NJ, Petrosino JF, Orwoll ES. Successful collection of stool samples for microbiome analyses from a large community-based population of elderly men. Contemporary clinical trials communications. 2017;7:158-62.

4. Chiusolo P, Metafuni E, Paroni Sterbini F, Giammarco S, Masucci L, Leone G, et al. Gut Microbiome Changes after Stem Cell Transplantation. Blood. 2015;126(23):1953.

5. Staffas A, Burgos da Silva M, van den Brink MRM. The intestinal microbiota in allogeneic hematopoietic cell transplant and graft-versus-host disease. Blood. 2017;129(8):927-33.

6. Goodrich Julia K, Di Rienzi Sara C, Poole Angela C, Koren O, Walters William A, Caporaso JG, et al. Conducting a Microbiome Study. Cell. 2014;158(2):250-62.

7. Paramsothy S, Borody TJ, Lin E, Finlayson S, Walsh AJ, Samuel D, et al. Donor Recruitment for Fecal Microbiota Transplantation. Inflammatory Bowel Diseases. 2015;21(7):1600-6.

8. Hogue SR, Gomez MF, da Silva WV, Pierce CM. A Customized At-Home Stool Collection Protocol for Use in Microbiome Studies Conducted in Cancer Patient Populations. Microbial Ecology. 2019;78(4):1030-4.

9. Mikail M, O'Doherty KC, Poutanen SM, Hota SS. Ethical implications of recruiting universal stool donors for faecal microbiota transplantation. The Lancet Infectious Diseases. 2020;20(3):e44-e9.

10. Lecky DM, Hawking MKD, McNulty CAM, group Es. Patients' perspectives on providing a stool sample to their GP: a qualitative study. The British journal of general practice : the journal of the Royal College of General Practitioners. 2014;64(628):e684-e93.

11. Ma Y, Chen H, Lan C, Ren J. Help, hope and hype: ethical considerations of human microbiome research and applications. Protein & cell. 2018;9(5):404-15.

12. Davis M, Oaten M, Occhipinti S, Chambers SK, Stevenson RJ. An investigation of the emotion of disgust as an affective barrier to intention to screen for colorectal cancer. European Journal of Cancer Care. 2017;26(4):e12582.

13. Green BB, Anderson ML, Cook AJ, Chubak J, Fuller S, Kimbel KJ, et al. Financial Incentives to Increase Colorectal Cancer Screening Uptake and Decrease Disparities: A Randomized Clinical Trial. JAMA Network Open. 2019;2(7):e196570.

14. Mehta SJ, Feingold J, Vandertuyn M, Niewood T, Cox C, Doubeni CA, et al. Active Choice and Financial Incentives to Increase Rates of Screening Colonoscopy–A Randomized Controlled Trial. Gastroenterology. 2017;153(5):1227-9.e2.

15. Donaldson GW, Moinpour CM, Bush NE, Chapko M, Jocom J, Siadak M, et al. Physician Participation in Research Surveys:A Randomized Study of Inducements to Return Mailed Research Questionnaires. Evaluation & the Health Professions. 1999;22(4):427-41.

16. Haff N, Patel MS, Lim R, Zhu J, Troxel AB, Asch DA, et al. The Role of Behavioral Economic Incentive Design and Demographic Characteristics in Financial Incentive-Based Approaches to Changing Health Behaviors: A Meta-Analysis. American Journal of Health Promotion. 2015;29(5):314-23.

17. Mehta SJ, Pepe RS, Gabler NB, Kanneganti M, Reitz C, Saia C, et al. Effect of Financial Incentives on Patient Use of Mailed Colorectal Cancer Screening Tests: A Randomized Clinical Trial. JAMA Network Open. 2019;2(3):e191156.

18. Kullgren JT, Dicks TN, Fu X, Richardson D, Tzanis GL, Tobi M, et al. Financial Incentives for Completion of Fecal Occult Blood Tests Among Veterans: A 2-Stage, Pragmatic, Cluster, Randomized, Controlled Trial. Annals of Internal Medicine. 2014;161(10_Supplement):S35-S43.

19. Gupta S, Miller S, Koch M, Berry E, Anderson P, Pruitt SL, et al. Financial Incentives for Promoting Colorectal Cancer Screening: A Randomized, Comparative Effectiveness Trial. American Journal of Gastroenterology. 2016;111(11):1630-6.

# Supporting information

**S1 File. Data**

Fig 1

Click here to access/download;Figure;Fig1.tif

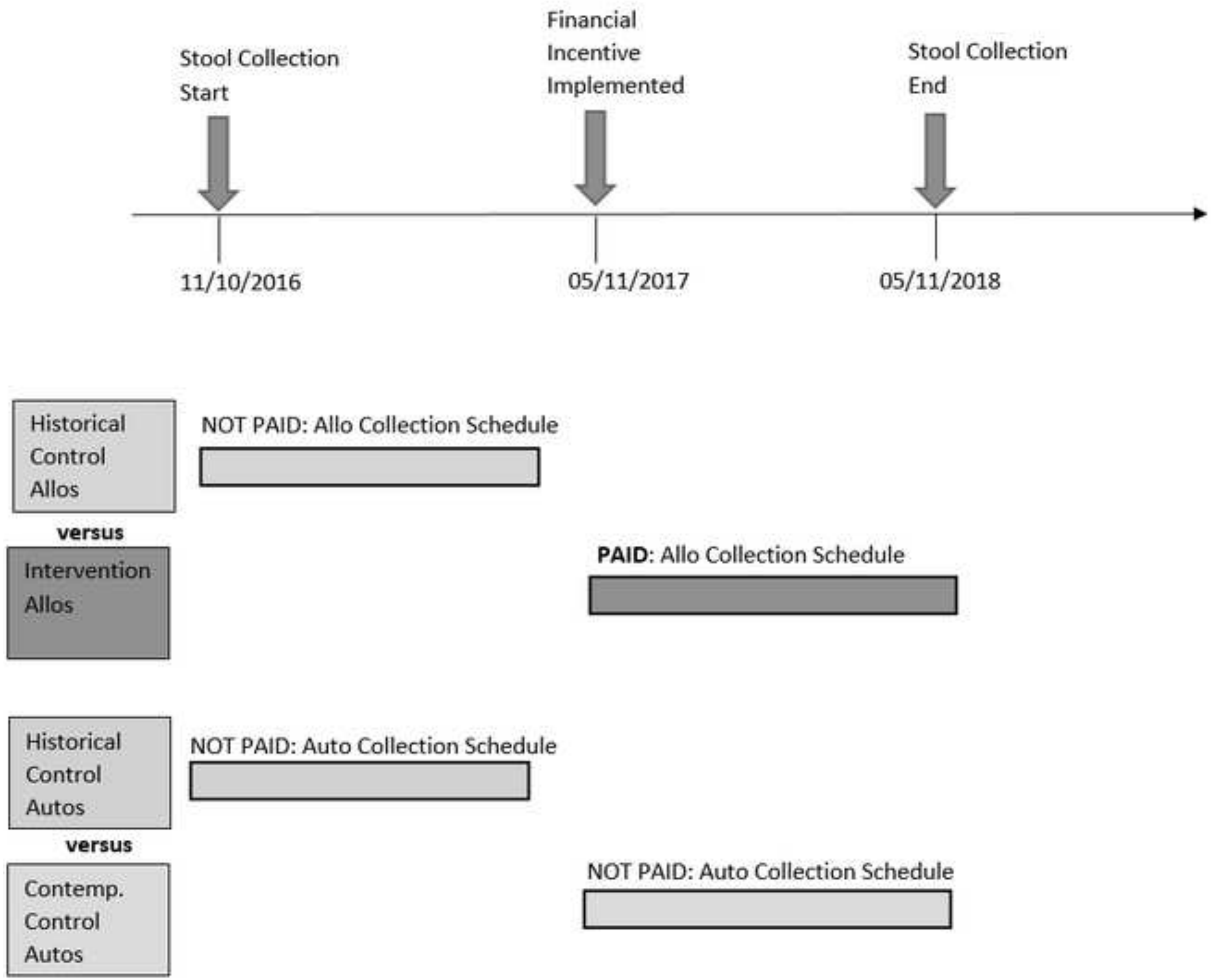

Fig 2a

Click here to access/download;Figure;Fig2a.tif

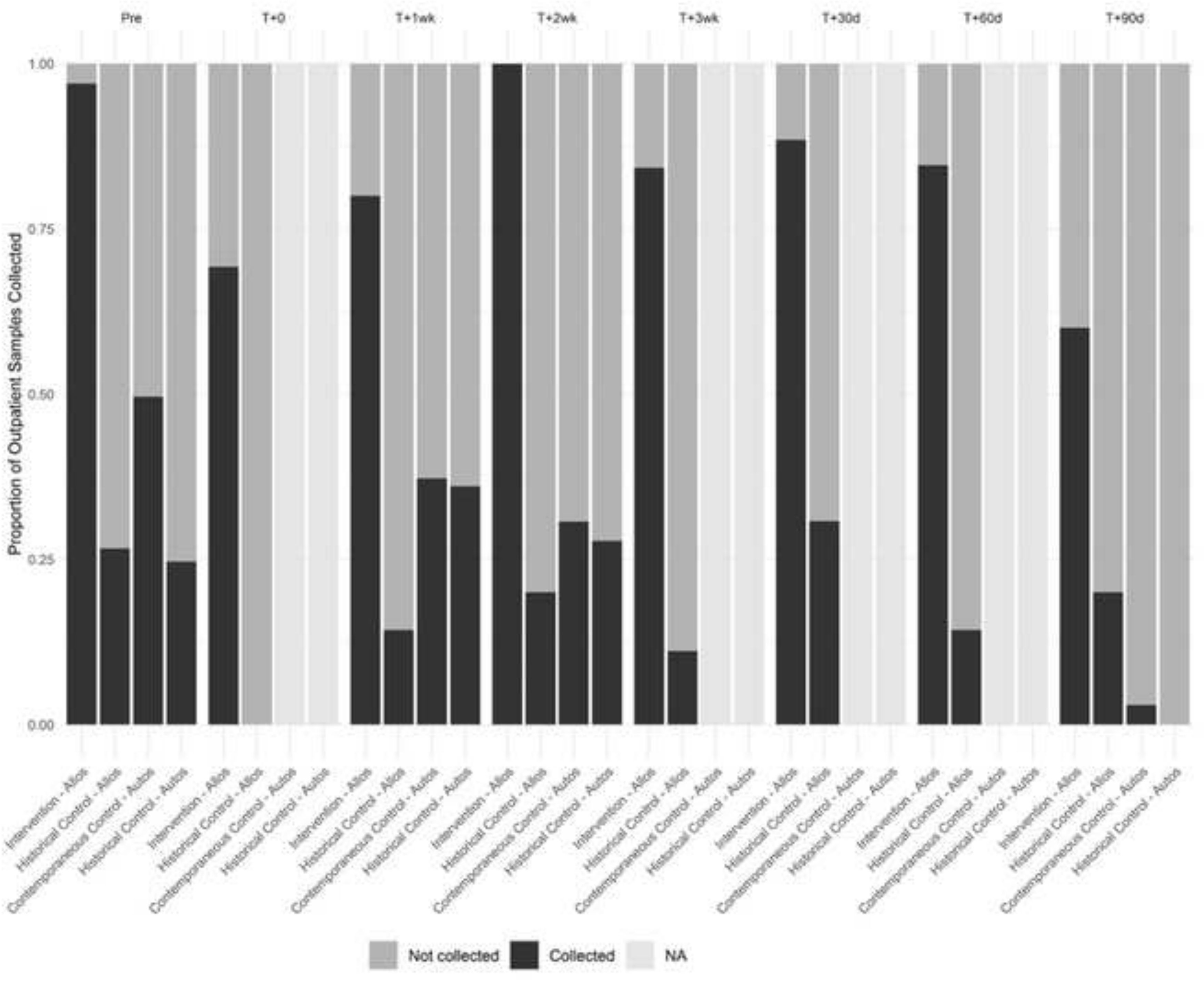

Fig 2b

Click here to access/download;Figure;Fig2b.tif

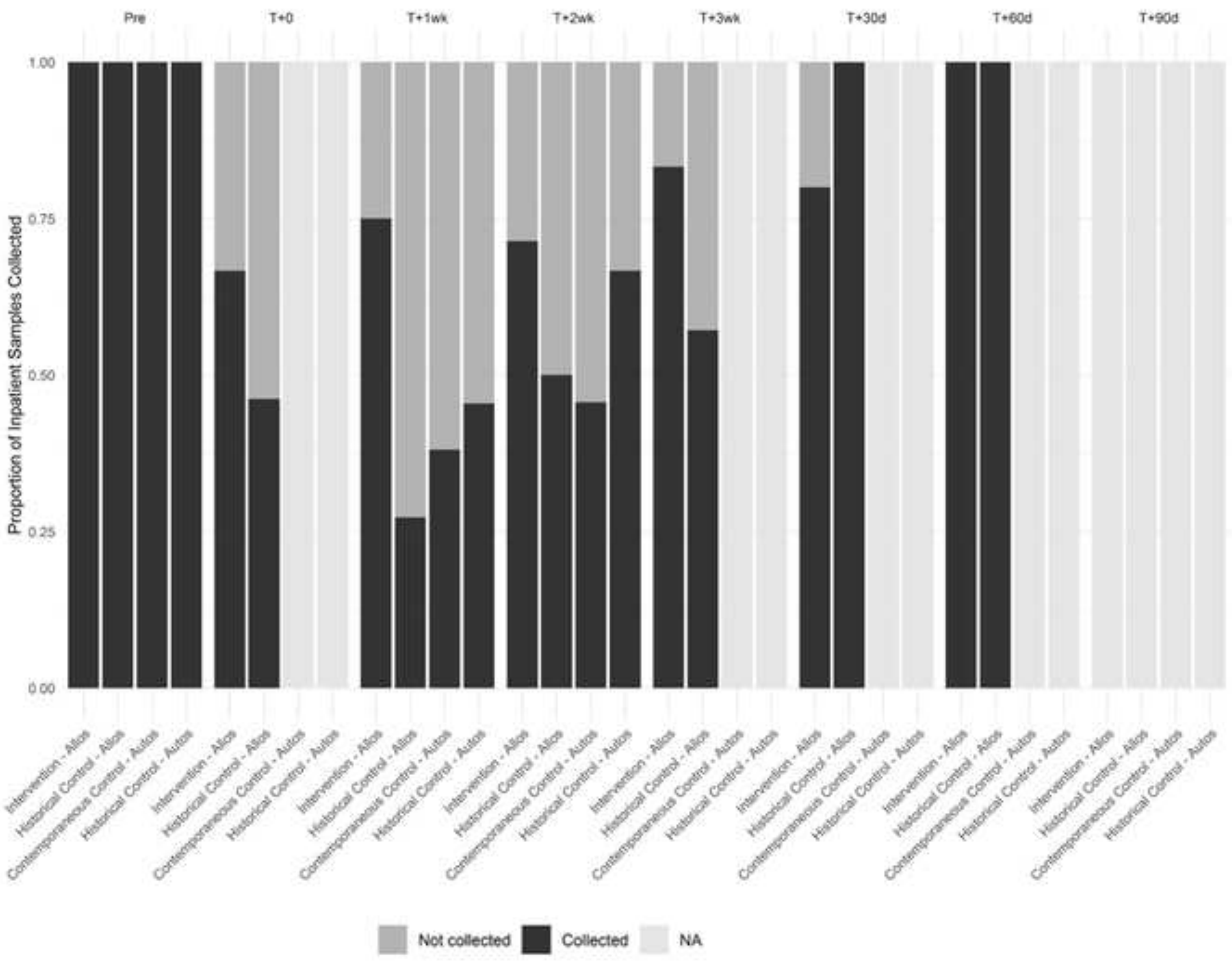

S1 File. Data

Click here to access/download
**Supporting Information**
S1 File.xlsx
