# Supplementary material for: Financial incentives to increase stool collection rates for microbiome studies in adult bone marrow transplant patients
Source: PLoS One. 2022 May 4;17(5):e0267974. doi: 10.1371/journal.pone.0267974 (PMC9067695; doi:10.1371/journal.pone.0267974)
Supplement: Attachment — Submitted filename: financial incentives_PLOSONE_Jan2022.docx [file pone.0267974.s003.docx]

The topic of financial incentives for patients is debated on grounds of both efficacy and ethics. A checklist was developed for healthcare professionals (Glasziou PP, Buchan H, Del Mar C, Doust J, Harris M, Knight R, Scott A, Scott IA, Stockwell A. When financial incentives do more good than harm: a checklist. BMJ. 2012 Aug 13;345:e5047. doi: 10.1136/bmj.e5047. PMID: 22893568.) and some of the items can also be relevant for financial incentives directed to patients:

1. Does the desired action improve patient outcomes?

#### 2. Will undesirable behaviour persist without intervention?

#### 3. Are there valid, reliable, and practical measures of the desired behaviour?

#### 4. Have the barriers and enablers to improving behaviour been assessed?

#### 5. Will financial incentives work, and better than other interventions to change behaviour, and why?

#### 6. Will benefits clearly outweigh any unintended harmful effects, and at an acceptable cost?

#### The authors undertook a controlled before after study to measure the effect of a $10 petrol gift card on the collection of stool samples for patients undergoing allogenic hematopoietic stem cell transplantation. I have some concerns about the research reported here, both technical and ethical.

#### General comments

#### I think a discussion on the appropriateness and ethics of the financial incentive in this context, based for example on the BMJ checklist and on the following article (Bartholomew T, Colleoni M, Schmidt H. Financial incentives for breast cancer screening undermine informed choice. BMJ. 2022 Jan 10;376:e065726. doi: 10.1136/bmj-2021-065726. PMID: 35012959.), would be welcome. Also I would question the opportunity of gift cards for petrol at a time when we are globally trying to reduce pollution. Why the choice of petrol and not vouchers for cultural goods as in BMJ 2021;375:e065217 for example?

#### Do patients derive a personal benefit from the microbiome study, in terms of treatment adaptation for example, or is it only for research that will eventually benefit other patients later?

#### For the primary endpoint, I would like an explanation of how it was calculated. What does a mean rate of 80% mean in Table 3, is it 80% of the population who brought the planned 8 stools? Or is it the total number of stools divided by 8 and by the total population? What is the most important for you research, to have the required number of samples for the population of to have as many patients as possible with at least one stool?

#### Specific comments:

#### Why is collection rate, and not the total number of stools collected chosen as the endpoint?

#### Also I am not sure that the term ‘rate’ is mathematically correct if you mean percentage. Is time a variable included in your rate calculations?

#### I understood from Fig 1 that 8 samples are planned. Why not report the average number of stools collected per patient and per period (before/after)?

#### Why is the total number of stools collected not reported in the auto group either? I saw that all the figures are I the excel file so it would be possible to present at least the endpoints that I think would be relevant:

#### % of patients with a full stool collection before/ after and in the control group.

#### % of the total number of samples collected (N collected / N population x8)

#### Why is there no sample size calculation?

#### The bar graphs figure 2 a and 2b are very difficult to read, I would suggest a figure like this, with line and an arrow indicating the introduction of the incentive in the alloHCT intervention group (https://qualitysafety.bmj.com/content/25/5/303)
