# Supplementary material for: Financial incentives to increase stool collection rates for microbiome studies in adult bone marrow transplant patients
Source: PLoS One. 2022 May 4;17(5):e0267974. doi: 10.1371/journal.pone.0267974 (PMC9067695; doi:10.1371/journal.pone.0267974)
Supplement: Attachment — Submitted filename: Response to reviewers_Thompson04-14-22.docx [file pone.0267974.s006.docx]

**Rebuttal letter**: Thank you very much for consideration of our manuscript, and we greatly appreciate your feedback and commitment to the peer review process. Please see our response to the reviewers’ comments. We have revised the paper accordingly.

04-14-2022

PONE-D-21-40817R1

Financial incentives to increase stool collection rates for microbiome studies in adult bone marrow transplant patients

PLOS ONE

Please include the following items when submitting your revised manuscript:

- A rebuttal letter that responds to each point raised by the academic editor and reviewer(s). You should upload this letter as a separate file labeled 'Response to Reviewers'.

**Response:** the rebuttal letter has been uploaded.

- A marked-up copy of your manuscript that highlights changes made to the original version. You should upload this as a separate file labeled 'Revised Manuscript with Track Changes'.

**Response:** 'Revised Manuscript with Track Changes' has been uploaded.

- An unmarked version of your revised paper without tracked changes. You should upload this as a separate file labeled 'Manuscript'.

**Response:** Manuscript has been uploaded.

**Comments to the Author**

1. If the authors have adequately addressed your comments raised in a previous round of review and you feel that this manuscript is now acceptable for publication, you may indicate that here to bypass the “Comments to the Author” section, enter your conflict of interest statement in the “Confidential to Editor” section, and submit your "Accept" recommendation.

Reviewer #1: (No Response)

Reviewer #2: All comments have been addressed

2. Is the manuscript technically sound, and do the data support the conclusions?

The manuscript must describe a technically sound piece of scientific research with data that supports the conclusions. Experiments must have been conducted rigorously, with appropriate controls, replication, and sample sizes. The conclusions must be drawn appropriately based on the data presented.

Reviewer #1: Partly

Reviewer #2: Yes

3. Has the statistical analysis been performed appropriately and rigorously?

Reviewer #1: No

Reviewer #2: Yes

4. Have the authors made all data underlying the findings in their manuscript fully available?

The [PLOS Data policy](http://www.plosone.org/static/policies.action#sharing) requires authors to make all data underlying the findings described in their manuscript fully available without restriction, with rare exception (please refer to the Data Availability Statement in the manuscript PDF file). The data should be provided as part of the manuscript or its supporting information, or deposited to a public repository. For example, in addition to summary statistics, the data points behind means, medians and variance measures should be available. If there are restrictions on publicly sharing data—e.g. participant privacy or use of data from a third party—those must be specified.

Reviewer #1: Yes

Reviewer #2: Yes

5. Is the manuscript presented in an intelligible fashion and written in standard English?

PLOS ONE does not copyedit accepted manuscripts, so the language in submitted articles must be clear, correct, and unambiguous. Any typographical or grammatical errors should be corrected at revision, so please note any specific errors here.

Reviewer #1: Yes

Reviewer #2: Yes

6. Review Comments to the Author

Please use the space provided to explain your answers to the questions above. You may also include additional comments for the author, including concerns about dual publication, research ethics, or publication ethics. (Please upload your review as an attachment if it exceeds 20,000 characters)

Reviewer #1: Please add in the statistical analysis section the sentence written in your answers to reviewers:
'A priori sample size calculation was not performed for this study. While this study was
approved by the Duke IRB and written informed consent was obtained from all study participants,
this study was not formally planned. '

**Response:** The requested information has been added to the statistical analysis of the methods section.

Reviewer #2: The author has addressed issues raised in the comments and greatly improved the quality and soundness of paper, therefore, I recommend the paper to be published.

**Response:** Thank you.
